# An Electronic Teaching Module for Improving Knowledge of Self-Management of Vaso-Occlusive Pain Crises in Patients With Sickle Cell Disease: Pilot Questionnaire Study

**DOI:** 10.2196/13501

**Published:** 2019-06-20

**Authors:** Tammie Tam, Maria R Baer, Lewis L Hsu, Jennie Y Law

**Affiliations:** 1 University of Maryland School of Medicine University of Maryland Baltimore, MD United States; 2 Marlene and Stewart Greenebaum Comprehensive Cancer Center University of Maryland School of Medicine University of Maryland Baltimore, MD United States; 3 Comprehensive Sickle Cell Center Division of Pediatric Hematology/Oncology, Department of Pediatrics University of Illinois at Chicago Chicago, IL United States

**Keywords:** patient education, teaching materials, portable electronic applications, sickle cell disease

## Abstract

**Background:**

For patients with sickle cell disease (SCD), effective management of vaso-occlusive crises (VOCs) is integral to provision of care, as nearly all affected individuals will suffer from VOCs in their lifetime. A recent systematic review of technological interventions to improve self-management in the care of SCD concluded that electronic health has the potential to improve the care of individuals with SCD.

**Objective:**

The aim of this study was to assess the value of an electronic teaching module (ETM) provided by Emmi Solutions for educating adult SCD patients on VOC self-management and treatment options for SCD.

**Methods:**

A pretest assessed adults with SCD for baseline knowledge with regard to self-management of VOCs. Participants then watched the 35-min ETM and completed a posttest and survey on the ETM.

**Results:**

A total of 20 adults enrolled. Their knowledge scores improved (pretest median 66.5% and posttest median 85%; *P*<.001). In total, 18 participants (18/20, 90%) agreed that they “learned a lot” or “learned something” from the ETM. The most common topic about which they reported learning was hydroxyurea. A total of 12 participants (12/20, 60%) agreed with the statement that they “would recommend the module to a friend or family member with sickle cell disease.”

**Conclusions:**

The ETM is associated with an increase in knowledge in patients with SCD. Limitations of the study include small sample size, no assessment of knowledge before premodule questionnaire completion, and no longitudinal follow-up. Identifying patients with SCD who demonstrate affinity for self-education via an ETM may further enhance utility of this tool to educate and empower patients.

## Introduction

### Background

For patients with sickle cell disease (SCD), effective management of vaso-occlusive crises (VOCs) is integral to provision of care, as nearly all affected individuals will suffer from VOCs in their lifetime [1]. Unfortunately, many patients with SCD lack access to specialized hematology care and, as a result, are reliant on providers who may be unaware of the myriad complications related to this chronic condition and may be uncomfortable with treating these patients [2]. Given the limited number of sickle cell day hospitals, many adult patients rely heavily on acute care services for management of their VOCs, despite often facing ineffective analgesic management and patient-perceived biases in care provision in the emergency department [3,4]. Provider desire for clinical correlates to confirm the complaint of pain in a patient with SCD leads to disbelief or skepticism of the patient and ultimately contributes to inadequate treatment of VOCs, resulting in premature discharges as well as high rates of emergency department and hospital recidivism [5,6]. In recent studies, patients with SCD report inadequate access to ambulatory care, poor communication with health care professionals, and lack of involvement in decisions regarding their own health [1,5]. Acute care encounters and rehospitalizations are frequent for patients with SCD, particularly for adolescents and young adults (AYAs) aged 18 to 30 years [7]. Given this gap in the knowledge and comfort level of health care providers managing patients with SCD, there is a clear need to improve patient health literacy and enhance patient confidence in self-care [8].

Electronic health (eHealth) may especially resonate with AYA patients, given their generational predilection for electronic media. A recent study showed that almost all AYA patients with SCD had daily internet access via either computers (84%) or mobile phones (70%) [9]. In one study examining self-management tools in SCD, the majority of participants opted to go online for their health information and preferred websites with interactive or social features (83%) [7]. Harnessing the power of Web-based modules to engage a vulnerable patient population is critical, given the positive correlation between patient confidence in self-care and improved health outcomes for individuals with SCD [8].

A recent systematic review of technological interventions to improve self-management in the care of SCD concluded that eHealth has the potential to improve the care of individuals with SCD [10]. eHealth is being used for self-management of a variety of chronic diseases, including SCD, asthma, diabetes, and hypertension [10]. Most eHealth intervention studies for SCD have been performed in children and adolescents [10]. Relatively few studies have evaluated eHealth in adults with SCD and they focus on pain or knowledge about reproductive health [10]. More studies are needed in adults with SCD in other components of care, including medication adherence, coping strategies, and clinic appointment adherence such as eHealth in pediatric SCD [10].

### Objectives

Given the lack of evaluation of eHealth interventions in adults, the purpose of this pilot study was to assess the value of the electronic teaching module (ETM) produced by Emmi Solutions for self-management in individuals with SCD. The ETM is an educational video with graphics and audio written at a fifth-grade reading level, with the exception of key words such as hemoglobin and hydroxyurea. It includes information about SCD and how self-care can help mitigate VOC triggers and decrease emergency department usage; and it encourages utilization of specialized care with a hematologist and coping strategies to manage pain at home with both pharmacologic and nonpharmacologic measures. Topics covered in the ETM include an overview of basic SCD pathophysiology, SCD pain, over-the-counter medications, opioids, pain management tools, hydroxyurea, and when to call your doctor. Currently, management of SCD pain focuses on alleviation of symptoms with fluids, anti-inflammatory agents, and opioid analgesics [3,8]. As much of the initial management of early VOCs could be performed at home, the Emmi ETM is an eHealth intervention to make self-care information more accessible to patients with SCD. Specifically, this ETM may be especially helpful for individuals with SCD who lack access to a specialized sickle cell clinic, and the format should be a good fit for AYA patients with SCD who prefer to use electronic methods to receive health information. We hypothesized that there would be an improvement in scores from the premodule knowledge-based questionnaire to the postmodule questionnaire and that the postmodule survey would indicate that the ETM is well-received by patients with SCD.

## Methods

### Measures

The pre- and postmodule knowledge-based questionnaires included 16 identical questions about self-management of sickle cell pain. The postmodule survey added 12 questions assessing participants’ satisfaction with use of the ETM and documenting age, gender, and highest level of education attained. The questionnaire (Multimedia Appendix 1) and survey (Multimedia Appendix 2) were written based on the Emmi ETM script by a professional education specialist from Emmi Solutions to assess for participant understanding of the content of the ETM. The pre- and postquestionnaire and survey responses were digitally recorded through SurveyMonkey, scored, and reviewed.

### Sample

A convenience sample of adults with SCD seen at the University of Maryland Medical Center (UMMC):

- Inclusion criteria: (1) Any type of SCD (HbSS, HbSβ0-thal, HbSC, or HbSβ+-thal), (2) aged 18 years or older, and (3) willing to commit 1.5 hours to complete the study in one sitting.
- Exclusion criteria: (1) unable to read English or (2) previous use of the Emmi ETM.

### Procedures

A convenience sample of patients with SCD was offered enrollment upon presentation to their scheduled Hematology outpatient visits at the UMMC between June and October 2017. After verbal consent, patients were provided with a laptop computer to view the Emmi ETM and complete the assessments in the clinic. Participants completed a pretest assessing their baseline knowledge with regard to self-management of VOCs. They then watched a 35-min ETM that described self-care techniques that can mitigate triggers for vaso-occlusion and decrease emergency department usage and also reviewed pharmacologic and nonpharmacologic measures for pain management at home. Patients were allowed to take breaks if necessary. It was noted if the participant fell asleep during the ETM. Participants then completed a posttest assessing their knowledge of self-management of VOCs and a survey regarding use of the ETM. The pre- and postmodule knowledge-based questionnaires and postmodule survey were deidentified to prevent a possible breach of privacy. Once participants completed the study, they received a US $20 Target^TM^ gift card for the time invested in the study. They were also given an information sheet about the study with their identification number and a link to re-access the ETM. This study was approved by the University of Maryland School of Medicine Institutional Review Board.

### Data Analysis

Pre- and postmodule questionnaire scores were compared using paired *t* tests. Postmodule survey responses were analyzed by frequency distribution. Data were monitored to see whether patients re-accessed the ETM.

## Results

A total of 39 individuals were approached for the study: 19 declined to participate (most stating reluctance to invest the amount of time needed), none were ineligible, and 20 participated. In total, 12 patients (12/20, 60%) were female. The median age of participants was 27.5 years (range: 20 to 48 years). The highest levels of education completed were as follows: not graduated high school (3), high school diploma/Generalized Educational Development (12), Bachelor’s degree (2), and beyond Bachelor’s degree (3).

There was significant improvement in scores from the premodule questionnaire to the postmodule questionnaire. The median pretest knowledge score was 66.5% and the median posttest knowledge score, after completion of the ETM, was 85% (paired *t* test [n=20]=6.84; *P*<.001; Table 1). This suggests that study participants were able to learn from the ETM and improve their baseline knowledge regarding self-management of sickle cell pain. The small sample size precludes a meaningful analysis of subgroups.

**Table 1:** Premodule versus postmodule questionnaire scores.

Study participant	Premodule Score (%)	Postmodule Score (%)	Change (%)
1	65	62	–4.6
2	85	97	14.1
3	68	97	42.6
4	56	85	51.8
5	76	85	11.8
6	62	85	37.1
7	74	74	0.0
8	47	59	25.5
9	65	94	44.6
10	79	88	11.4
11	65	71	9.2
12	59	82	39.0
13	38	47	23.7
14	56	82	46.4
15	74	88	18.9
16	71	88	23.9
17	51	74	45.1
18	74	79	6.8
19	68	94	38.2
20	88	97	10.2
Average	66.1	81.4	24.8

**Figure 1:**
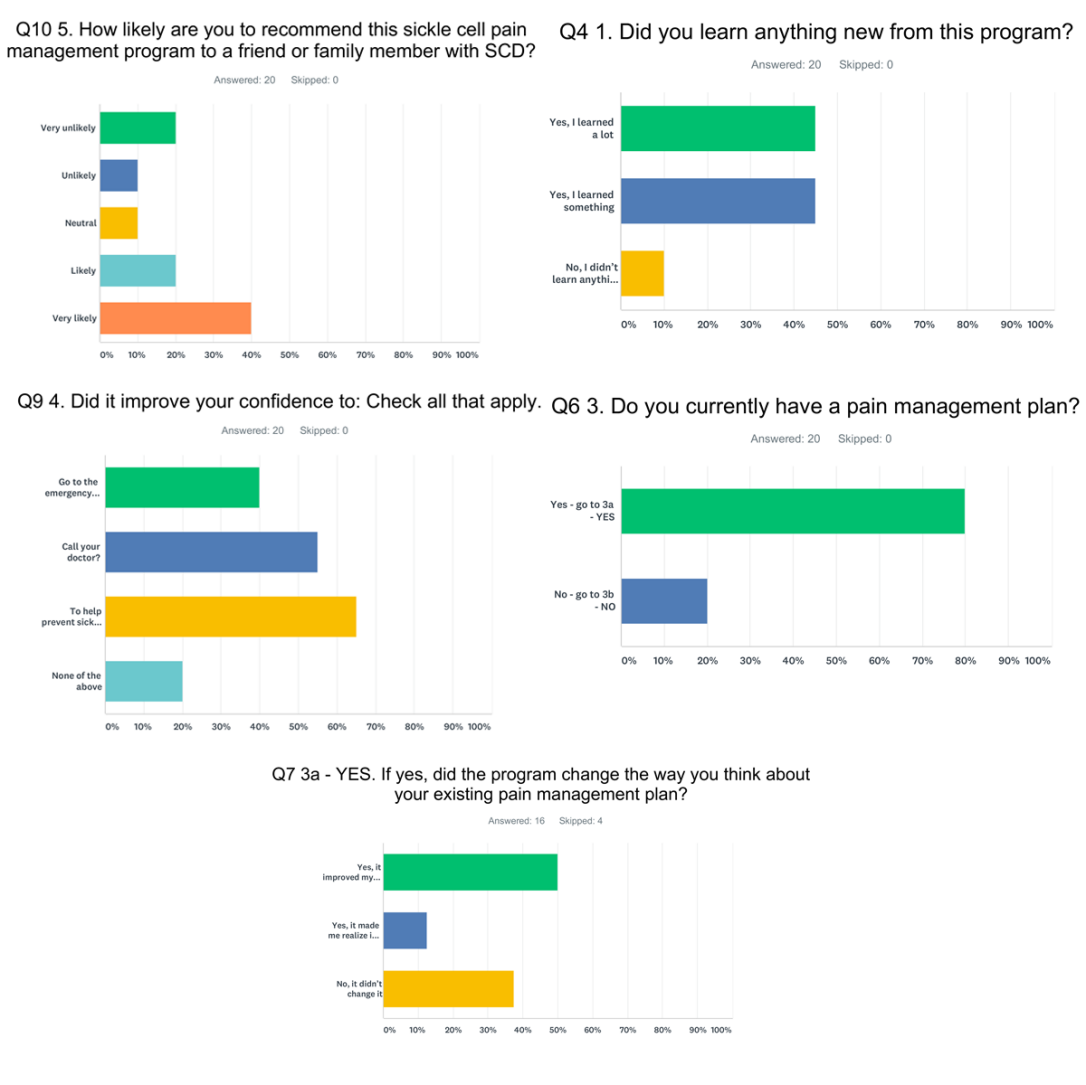
Excerpts from postmodule survey responses. SCD: sickle cell disease.

The postmodule survey (Figure 1) results indicated that the ETM was well-received. In total, 18 out of 20 participants (90%) reported that they *learned a lot* or *learned something* from the ETM. The most common topic about which they reported learning was hydroxyurea. Nine of the 20 participants commented that they gained a better understanding of how hydroxyurea works and/or of its side effects. Five participants also commented that they learned a lot about opioids. Sixteen of the 20 participants also reported that the ETM improved their confidence to help prevent sickle cell crises, call their doctor, and/or go to the emergency department when needed. Sixteen of the 20 participants had an existing pain management plan, as expected as all study participants were scheduled to see sickle cell specialists in the adult Hematology clinic at UMMC. Of the 16 participants with a pain management plan, 2 reported that the ETM made them realize that they needed to revisit or update their plan with their doctor, 8 reported that the ETM improved their confidence to manage pain, and 6 reported that the ETM had no effect on the way they thought about their existing plan. Of the 4 participants who did not have an existing pain management plan, all 4 reported that they were very likely to ask their doctor or pain specialist to create a pain management plan with them in the next 3 months. Twelve of the 20 participants (60%) were either *very likely* or *likely* to recommend the ETM to a friend or family member with SCD.

Five participants fell asleep at some point during the ETM, which at least 3 patients attributed to fatigue. One participant commented that the 25-min ETM was “way too long, especially if the target audience is users with this condition” *.* Patient access to the ETM was monitored for 6 months after the completion of the study and no patients re-accessed the ETM.

## Discussion

### Principal Findings

The knowledge scores showed a significant improvement from the premodule questionnaire to the postmodule questionnaire. In the postmodule survey, 90% of participants reported that they learned from the ETM, and 60% of participants were likely to recommend the ETM to others.

The small sample size precludes a meaningful analysis of subgroups defined by factors including age, sex, and level of education. The subgroup analysis might have shown differences between age groups owing to the generational predilection for electronic media and differences between the level of education subgroups owing to varying levels of comprehension of the ETM or the questionnaire. For future studies with a larger sample size, a literacy assessment might be a more accurate measure of study participants’ comprehension of the ETM than the self-reported level of education. The disadvantage of adding a literacy assessment to the study is that some patients might not have the attention span to concentrate for a longer study.

Subgroups of patients who may not have benefited as much from the Emmi ETM include those who had barriers to completing the ETM, such as a low level of computer literacy, reading level below fifth grade, and short attention span, and those with a high baseline knowledge of self-care before the Emmi ETM intervention. Falling asleep during the module may be attributable to fatigue, a well-known complication of SCD that can be out of proportion to the anemia [11,12]. Although the 2 participants who reported that they did not learn anything new from the ETM scored 85% and 71% on the premodule questionnaire, both improved on the postmodule questionnaire, with postmodule scores of 97% and 88%. Other participants who scored >70% on the premodule questionnaire reported that they did learn something from the ETM.

Given that 5 participants fell asleep at some point during the ETM and no patients re-accessed the ETM within 6 months of the study, revisions may be necessary. In a recent study assessing online module development, students were more engaged when the module content was applicable to their personal life or community and provided opportunities for critical thinking [13]. The study also highlights the value of emphasizing qualitative feedback over traditionally quantitative feedback [13]. Possible changes to make the ETM more engaging include adding a playback speed control button per study participant survey comments, modifying the ETM to make it more interactive by adding questions after each section and adding a summary at the end of the ETM. In addition, we could implement an exit interview instead of an online survey to more rigorously evaluate how patients felt about the ETM. Many patients were noted to be slow at typing, so a verbal survey may encourage a more lengthy and critical assessment of the ETM.

Although the goal of health education is to demonstrate clinical benefit, this demonstration will require additional investigations beyond the scope of this pilot study. Next steps include expanding the study to include a larger sample size and multiple sites (urban vs rural and medical vs community locations). A recent systematic review concluded that evaluations of SCD educational interventions should consist of large-scale studies with many patients retained in a longitudinal follow-up, owing to the heterogeneity of SCD and the modest effect size for educational interventions [14].

### Limitations

Limitations of this study include a small sample size, no assessment of literacy, no validation of the questionnaire, no assessment of patient knowledge of SCD before the initial premodule questionnaire completion, and a lack of longitudinal follow-up to track retention of knowledge gained from the ETM or further actions taken based on what participants learned from the ETM.

### Conclusions

Assessment of this pilot implementation of an educational ETM for SCD in a real clinical setting revealed both benefits and drawbacks. Nearly all patients gained some knowledge from the ETM and had positive comments such as willingness to recommend the ETM to friends. The wide range of pretest scores shows that, before the ETM, there are probably subgroups of patients with greater knowledge of self-care for SCD. The patients’ comments suggest that subgroups exist with different learning styles, with some requesting a more customized or streamlined ETM. Future studies should also examine whether the knowledge acquired through this ETM has an impact on disease course, although these will be difficult to conduct given the unpredictable nature of SCD. Finally, the high rate of refusal to participate (19 of 39 people approached for the study) suggests that modifications are needed to motivate people to use the ETM. Other modifications that may decrease the high rate of refusal to participate include giving patients the option to schedule the study visit at their convenience and having a trusted provider endorse the study.

### Practice Implications

The educational ETM has the potential to provide an immediate impact on health literacy for an underserved patient population and potentially improve disease management and disease-specific outcomes. Identifying discrete populations of patients with SCD who demonstrate an affinity for self-education via the Emmi ETM would further enhance the utility of this tool. Innovative approaches to improving self-care for patients with SCD are sorely needed and have the potential to enhance care provision for affected individuals, as well as transform the oftentimes adversarial interaction between adult patients and their treating physicians.

As more patients with SCD are reaching adulthood and SCD has a lifelong course, providing resources to facilitate the transition from pediatric to adult SCD care is essential. Early adulthood is a high-risk period, associated with increased mortality [8,9]. The ETM may be particularly helpful in facilitating the often-fraught transition from pediatric to adult care. Barriers to successful transition include AYA-specific patient issues (lack of knowledge about SCD and about obtaining SCD care with adult health care providers, lack of financial independence and decision-making experience, and fear of leaving familiar clinics), health care provider issues (poorly coordinated transitional care and lack of education and familiarity with SCD treatment), and systemic issues (adult care with fewer interdisciplinary services than well-established pediatric SCD programs, AYAs losing or changing insurance coverage, and a reimbursement system that may limit the willingness of specialists to see adult SCD patients) [15]. In addition, AYA patients may be expected to have a particular affinity for a Web-based educational tool.

## Appendix

Multimedia Appendix 1 Pre- and postmodule knowledge-based questionnaire.

Multimedia Appendix 2 Postmodule survey.

## Abbreviations

AYA: adolescent and young adult
eHealth: electronic health
ETM: electronic teaching module
SCD: sickle cell disease
UMMC: University of Maryland Medical Center
VOCs: vaso-occlusive crises
