# Supplementary material for: An Electronic Teaching Module for Improving Knowledge of Self-Management of Vaso-Occlusive Pain Crises in Patients With Sickle Cell Disease: Pilot Questionnaire Study
Source: JMIR Mhealth Uhealth. 2019 Jun 20;7(6):e13501. doi: 10.2196/13501 (PMC6610466; doi:10.2196/13501)
Supplement: Multimedia Appendix 1 [file mhealth_v7i6e13501_app1.docx]

**Sickle Cell Disease (SCD) Pain Management Knowledge Questions**

* = correct answer

1. **In sickle cell disease (SCD), why do some red blood cells cause problems?
   Check all that apply.** [Script 75-81]

⃞ They are too stiff and can get stuck in blood vessels*

⃞ They become sticky and can cause blockages*

⃞ They are smaller, can move too fast through blood vessels, causing pain

1. **Why does SCD sometimes cause joint pain in places like the hip or shoulder?
   Check all that apply.** [Script 114-120]

⃞ People with SCD are born with weaker joints (hips, knees, shoulders)

⃞ When the bones there don’t get enough blood and oxygen, part of the bone dies*

⃞ As people with SCD age, they make too much hemoglobin, which destroys the joints

⃞ Red blood cells are stickiest in the hip and shoulder areas, which makes pain more likely

1. **If there’s not enough blood and oxygen traveling through the body, it can cause real problems for the spleen. What other organs could be affected?
   Check all that apply.**[Script row 84]

⃞ Brain*

⃞ Heart*

⃞ Kidney*

⃞ Liver*

⃞ Skin*

⃞ Tongue

**4. When people with SCD have liver problems, they should NOT:
Check all that apply.**[SCD script 156-159]

⃞ Take aspirin

⃞ Take pills that contain ibuprofin, like Advil

⃞ Take pills that contain acetaminophen, like Tylenol*

⃞ Take pills with food

1. **Doctors help individuals figure out how much over-the-counter pain medication (like aspirin, Advil, and other NSAIDs) they should take each day. Why should people AVOID taking more NSAIDs without talking to their doctor first?
   Check all that apply.** [Script 152, 159, 168-171]

⃞ Too much can damage the kidneys*

⃞ Too much can damage the liver*

⃞ Too much can cause problems with bleeding*

⃞ Too much can cause dehydration

⃞ Too much can hurt the lining of the stomach*

1. **What problems should opioids be used to help treat?
   Check all that apply.** [Script 231-236]

⃞ Anxiety from SCD
⃞ Depression from SCD

⃞ Pain from an SCD crisis*

⃞ Long-term (chronic) pain*

⃞ Trouble falling asleep

1. **When it comes to sickle cell disease and fluids, it can help to:** [Script 349-353]
   **Check all that apply.**

⃞ Avoid drinks that contain caffeine*

⃞ Drink more water*

⃞ Always drink things with ice

1. **To avoid experiencing withdrawal from opioids, it’s best to:** [Script 250]
   **Just check ONE answer.**

⃞ Immediately stop taking them as soon as your pain is managed

⃞ Take them as often as you like

⃞ Take the smallest amount possible to manage the pain*

⃞ Try to build up a tolerance for opioids

1. **Which of these is TRUE? Check all that apply.** [Script 246-249]

⃞ If I’m in a lot of pain, I should take the highest dose of opioids possible to help

⃞ If I’ve taken opioids for a while, my body can build up a tolerance to them*

⃞ Needing a higher dose of opioids to control my pain is the same as being addicted to them

1. **When people take opioids for a while, their body gets used to them.** [Script 246-249]

**Check all that apply.**

⃞ This is opioid addiction

⃞ This is why they may need a higher dose to manage their pain*

⃞ This is why people feel sick if they suddenly stop taking them*

⃞ When this happens people can no longer use opioids

1. **Which of these make opioid addiction more likely?** [Script 269-273]

**Check all that apply.**

⃞ If someone in your family has addiction issues*

⃞ If you are currently taking NSAIDs or any over the counter pain medication

⃞ If you’ve had problems with addiction in the past*

⃞ If you’ve had problems with withdrawal from opioids in the past

⃞ If you have SCD

1. **If you go to an Emergency Department for help with SCD pain you should:** [Script 253-267]

**Check all that apply.**

🞎 Be able to get a refill on any opioid medication prescriptions

🞎 Bring medications with you from home in their original bottles*

🞎 Call your doctor before you go in*

🞎 Expect them to have access to your records from other EDs you’ve visited

🞎 Go to a different ED each time so they don’t think you’re a drug seeker

🞎 Have a family member or friend go with you*

1. **Hydroxyurea can help prevent sickle cell pain because:** [Script 394-401]
   **Check all that apply.**

⃞ It creates new red blood cells that are still sickle shaped, but softer

⃞ It helps the body make fetal hemoglobin (baby blood) again*

⃞ It increases the number of white blood cells the body makes

1. **Hydroxyurea is a common medication to make sickle cell disease less severe. Hydroxyurea can help prevent:** [Script 384-388]

**Check all that apply.**

⃞ Acute chest syndrome*

⃞ Avascular necrosis (bone death)

⃞ The need for blood transfusions*

⃞ Pain crises*

**15. When taking hydroxyurea for SCD:** [Script 402-413]

**Check all that apply.**

⃞ People often experience hair loss

⃞ People usually start taking a very small dose*

⃞ It’s usually taken as a shot (injection)

⃞ Women should make sure they do NOT get pregnant*

1. **Which of these can be side effects of hydroxyurea?** [Script 407-411]

**Check all that apply.**

⃞ A mild rash*

⃞ Feeling sick to your stomach*

⃞ Feeling thirsty

⃞ Hemorrhoids

Pre: https://www.surveymonkey.com/r/premodule

Post: https://www.surveymonkey.com/r/postmodule
