# Supplementary material for: An Electronic Teaching Module for Improving Knowledge of Self-Management of Vaso-Occlusive Pain Crises in Patients With Sickle Cell Disease: Pilot Questionnaire Study
Source: JMIR Mhealth Uhealth. 2019 Jun 20;7(6):e13501. doi: 10.2196/13501 (PMC6610466; doi:10.2196/13501)
Supplement: Multimedia Appendix 2 [file mhealth_v7i6e13501_app2.docx]

**Emmi Sickle Cell Self-Care Module Satisfaction questions**

**1.**     **Did you learn anything new from this program?**

⃞ Yes, I learned a lot

⃞ Yes, I learned something

⃞ No, I didn’t learn anything new

**2. If you learned something new, what did you learn? [free text] ______________________________**

**3. Do you currently have a pain management plan? Yes/No**⃞ If yes, go to 3a-yes

⃞ If no, go to 3a-no

**3a - YES. If yes, did the program change the way you think about your existing pain management plan?**

⃞ Yes, it improved my confidence it can help me manage pain

⃞ Yes, it made me realize i need to revisit or update it with my doctor

⃞ No, it didn’t change it

**3a - NO. If no, how likely are you to ask your doctor or pain specialist to create a pain management plan with you in the next 3 months?**

⃞ Very likely

⃞ Somewhat likely

⃞ Unlikely

⃞ Very unlikely

**4. Did it improve your confidence to:**

⃞ Go to the emergency department when needed?

⃞ Call your doctor?

⃞ To help prevent sickle cell crises?

**5.**     **How likely are you to recommend this sickle cell pain management program to a friend or family member with SCD?** [**https://www.netpromoter.com/know/**](https://www.netpromoter.com/know/)

⃞ Very unlikely

⃞ Unlikely

⃞ Neutral

⃞ Likely

⃞ Very likely

**6.**     **Do you have any thoughts or comments? (Free text) ______________________**

https://www.surveymonkey.com/r/emmisurvey
